# Effects of extremely low frequency pulsed magnetic fields on diabetic nephropathy in streptozotocin-treated rats

**DOI:** 10.1186/s12938-015-0121-6

**Published:** 2016-01-19

**Authors:** Feijiang Li, Tao Lei, Kangning Xie, Xiaoming Wu, Chi Tang, Maogang Jiang, Juan Liu, Erping Luo, Guanghao Shen

**Affiliations:** School of Biomedical Engineering, Fourth Military Medical University, Xi’an, China

**Keywords:** Pulsed magnetic fields, Diabetic nephropathy, Streptozotocin, rats

## Abstract

**Background:**

Extremely low frequency pulsed magnetic fields (ELFPMF) have been shown to induce Faraday currents and measurable effects on biological systems. A kind of very high frequency electromagnetic field was reported that it improved the symptoms of diabetic nephropathy (DN) which is a major complication of diabetes. However, few studies have examined the effects of ELFPMF DN at the present. The present study was designed to investigate the effects of ELFPMF on DN in streptozotocin (STZ)–induced type 1 diabetic rats.

**Methods:**

Adult male SD rats were randomly divided into three weight-matched groups: Control (non-diabetic rats without DN), DN + ELFPMF (diabetic rats with DN exposed to ELFPMF, 8 h/days, 6 weeks) and DN (diabetic rats with DN exposed to sham ELFPMF). Renal morphology was examined by light and electron microscopy, vascular endothelial growth factor (VEGF)-A and connective tissue growth factor (CTGF) were measured by enzyme linked immune sorbent assay.

**Results:**

After 6 weeks’ ELFPMF exposure, alterations of hyperglycemia and weight loss in STZ-treated rats with DN were not found, while both positive and negative effects of ELFPMF on the development of DN in diabetic rats were observed. The positive one was that ELFPMF exposure attenuated the pathological alterations in renal structure observed in STZ-treated rats with DN, which were demonstrated by slighter glomerular and tubule-interstitial lesions examined by light microscopy and slighter damage to glomerular basement membrane and podocyte foot processes examined by electron microscopy. And then, the negative one was that ELFPMF stimulation statistically significantly decreased renal expression of VEGF-A and statistically significantly increased renal expression of CTGF in diabetic rats with DN, which might partially aggravate the symptoms of DN.

**Conclusion:**

Both positive and negative effects of ELFPMF on the development of DN in diabetic rats were observed. The positive effect induced by ELFPMF might play a dominant role in the procession of DN in diabetic rats, and it is suggested that the positive effect should be derived from the correction of pathogenic diabetes-induced mediators.

## Background

Diabetic nephropathy (DN) is a major complication of diabetes [1]. Approximately 20–40 % of patients with type 1 or type 2 diabetes mellitus develop DN [2]. DN is the leading cause of chronic kidney disease accounting for nearly 50 % of all end-stage renal disease worldwide [3]. DN is characterized by increased glomerular permeability to proteins and excessive extracellular matrix accumulation in the mesangium, eventually resulting in glomerulosclerosis and progressive renal impairment [4].

The increasing prevalence of DN worldwide represents a major societal issue because of the enormous expense associated with kidney replacement therapy [5]. Current therapies that aim to lower blood glucose are not effective in blocking renal damage, and cotreatment with renoprotective drugs often results in potential toxicity, poor tolerability and ineffectiveness for some percent of diabetic patients [6]. Therefore, there is an urgent need to explore other non-pharmacological novel therapeutic modalities with efficacy and safety, particularly when patients with DN require a combined treatment with an oral renoprotective drug to preserve normal renal function and to prevent or slow the progression of DN.

Near infrared light is a kind of extremely high frequency electromagnetic wave, which has much higher frequency than ELFPMF. A study has reported that a near infrared light (670 nm low-level light) improved renal function and antioxidant defense capabilities in the kidney of streptozotocin (STZ)–induced type 1 diabetic rats [7]. Near infrared light and ELFPMF have very different effects in their interaction with living tissue, while ELFPMF is more widely studied than near infrared light in biological systems by our literature review. According to our literature review, ELFPMF has been shown to induce non-thermal effects on biological systems [8]. However, few studies have examined the effects of ELFPMF on DN at the present. Therefore, we aimed to investigate the effects of ELFPMF on kidney complications induced by diabetes.

In the current study, we used STZ-treated diabetic rats with DN to assess the effects of whole-body exposure to 15 Hz ELFPMF whose peak magnetic flux density (MFD) was approximate 1.6 × 10^−3^ T on the development of renal changes in DN by using morphological examination and ELISA analysis for VEGF-A and CTGF. The ELFPMF was generated by a self-produced apparatus and the exposure duration was 8 h per day, 6 days a week for 6 weeks. Our results demonstrate that ELFPMF had two-sided effects on renal damage induced by diabetes.

## Methods

### Experimental diabetes

Thirty-two adult male Sprague–Dawley rats, weighting 300 ± 20 g, were provided by Animal Center of the Fourth Military Medical University and housed in a room (Animal Center of the Fourth Military Medical University, Xi’an, China) with controlled temperature (23 ± 1 °C), relative humidity (50 ~ 60 %), and alternately light–dark cycle (12 h/12 h), with access to standard pellet and clean water. Eight rats were treated as non-diabetic control animals, and the rest 24 rats were used to induce diabetic models. Diabetes mellitus was induced by an intraperitoneal injection of STZ (55 mg/kg body weight) in freshly prepared citrate buffer (pH 4.5) after an overnight fast. Similarly, equivalent dose of sterile citrate buffer solution was injected into the control rats. Confirmation of hyperglycemia was made three days after STZ injection, and only STZ-treated rats whose glucose concentration of the tail venous blood measured by OneTouch SureStep Plus glucometer (Lifescan, Milpitas, CA, USA) were higher than 20 mmol/L were considered as qualified diabetic models [9]. Eight rats were excluded from the qualified diabetic models because of low blood glucose levels. The rest of rats were randomized into three weight-matched groups (eight in each group): non-diabetic rats without DN control group (Control), diabetic rats with DN exposed to ELFPMF group (DN + ELFPMF) which were subjected to whole-body exposure to 15 Hz ELFPMF 8 h (09:00–17:00) everyday, 6 days a week for 6 weeks and diabetic rats with DN exposed to sham ELFPMF exposure group (DN). Although the same ELFPMF apparatus was employed in DN group, the ELFPMF stimulator was not activated. ELFPMF stimulation was carried out the next day after confirmation of hyperglycemia. The current study was performed in adherence to the National Institutes of Health guidelines for the use of experimental animals, and all animal protocols were approved by the Committee for Ethical Use of Experimental Animals of the Fourth Military Medical University.

### ELFPMF apparatus

**Fig. 1:**
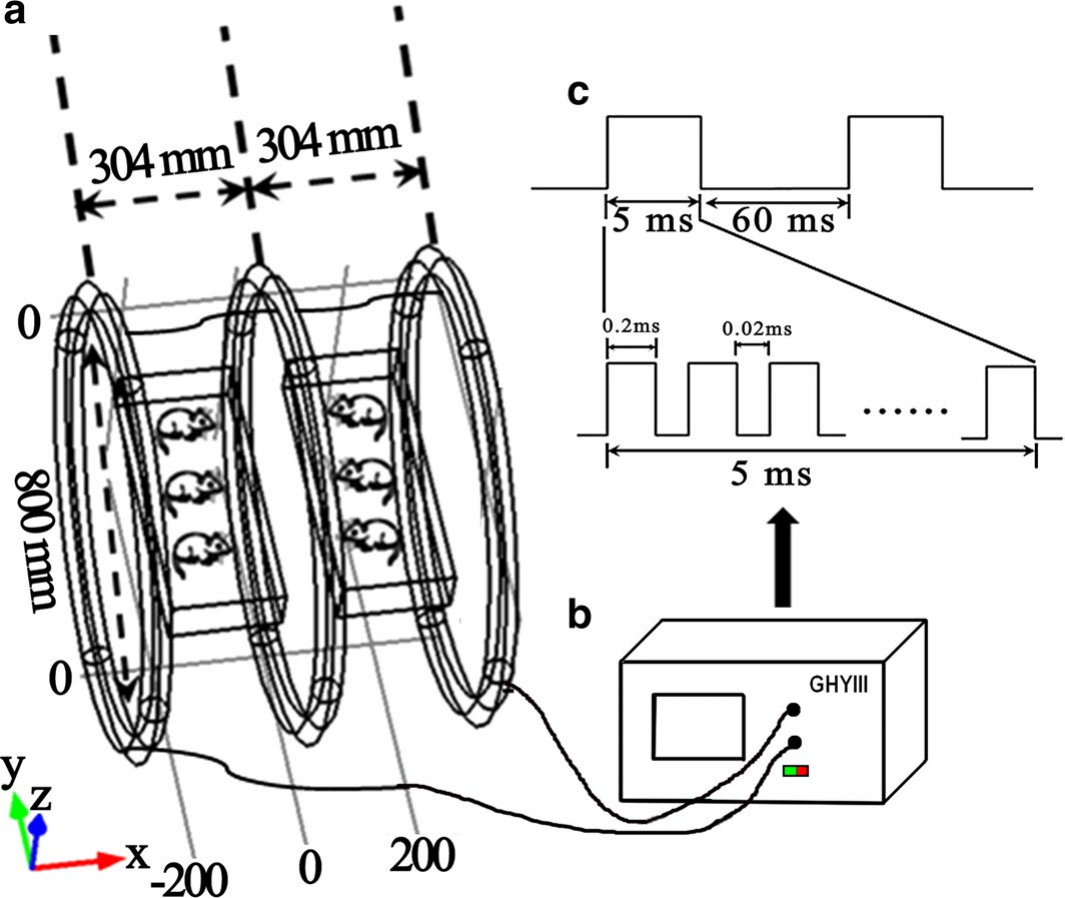
Schematic drawing of the ELFPMF apparatus and pulse protocol. **a** Modified Helmholtz coils assembled with three-coil array. **b** The pulsed signal generator GHY-III. **c** The open-circuit voltage waveform generated by GHY- III with a repetitive burst frequency at 15 Hz (burst width, 5 ms; burst wait, 60 ms; pulse width, 0.2 ms; pulse wait, 0.02 ms; pulse rise and fall time: 0.3, 2.0 μs)

The ELFPMF exposure system (GHY-III, FMMU, Xi’an, China; China Patent no.ZL02224739.4) was composed of a pulsed signal generator (Fig. 1b) and a modified Helmholtz coils assembled with three-coil array. This apparatus has presented has been described in detail in our previous study [10].

The modified Helmholtz coils was composed of three identical coils which were in series connection, with the same coil diameters of 800 mm and placed coaxially with a distance of 304 mm apart. Each coil was made up of enameled coated copper wire with 0.8 mm diameter. The number of turns of the central coil was 266, and the number of turns of the two outside coils was 500 (Fig. 1a). The modified Helmholtz configured with these parameters has presented significantly upgraded uniformity of MFD [10]. As depicted in Fig. 1c, the pulsed signal apparatus generated an open circuit waveform which was composed of a pulsed burst (burst width, 5 ms; burst wait, 60 ms; pulse width, 0.2 ms; pulse wait, 0.02 ms; pulse rise and fall time: 0.3, 2.0 μs) repeated at 15 Hz. This type of waveform has been proved effective in the prevention and treatment of bone disorders, cardiovascular and neurological diseases by our research group over a long period of time [10–12].

Two cubic plastic rat cages containing rats in DN + ELFPMF group were put in the center of every two neighboring coils (the length of the cage was along O–Y direction) and cages were supported by stands to let the activities of rats restrict on the XY plane which had higher intensity and better uniformity of MFD (Fig. 1). The distribution of the peak MFD was measured by using a Gaussmeter (Model 455 DMP Gaussmeter, Lake Shore Cryotronics, USA), and the measurement result was (1.6 ± 0.1) × 10^−3^ T (mean ± SD) in the exposure area (cage: 50 cm long, 20 cm wide and 15 cm high).

### Measurements of weights and blood glucose levels

The weights and blood glucose levels of all rats were evaluated prior to STZ administration, and there were no statistically significant differences for these parameters among three groups. In addition, weights and blood glucose levels of all rats were measured after 6-week ELFPMF exposure period.

### Get the tissue samples

Six weeks after ELFPMF stimulation in STZ-treated rats with DN, all rats were anesthetized by an intraperitoneal injection of 7 % chloral hydrate solution (0.45 ml/100 g) prior to collecting the kidney. Both kidneys for each rat were harvested and blotted. Left or right kidney for each rat was randomly chosen to carry morphological examination and ELISA analysis respectively.

### Light microscopy of renal morphology

The renal cortex samples were fixed by immersion in 10 % neutral buffered formalin. After being embedded in paraffin, 4 μm sections were stained with hematoxylin-eosin (HE), periodic acid Schiff (PAS), and Masson’s trichrome. Two sections for every staining method were got per rat, and five different sites of renal glomeruli were viewed per section. All the slides were examined by a renal pathologist (YW) in single blind fashion.

### Ultrastructural examination of renal glomeruli

The renal cortex was dissected and post fixed by immersion in the fixative solution (2 % paraformaldehyde, 2 % glutaraldehyde, 0.1 M cacodylate buffer at pH 7.3) for 2 h at 4 °C, and washed in 0.1 M cacodylate buffer, and osmicated for 4 h in 1 % OsO_4_ (Fluka). Sections of renal cortex were rinsed in 0.1 M cacodylate buffer, dehydrated and embedded in epoxy 812-Araldite (Polysciences). Ultra-thin sections (80 nm) were subsequently cut, collected on cellodincoated single slot grids and stained with uranyl acetate and lead citrate. The microphotographs were obtained using a transmission electron microscope (JEM-1230, Japan) operated at 80 keV, and the thickness of glomerular basement membrane (GBM) was measured using the ruler function of the software. Average GBM thickness for each rat was calculated from 30 measurements obtained from different sites of GBM, and eight rats were chosen to evaluate the thickness of GBM for each group.

### ELISA

After cutting kidney cortex samples, we checked the weight, added PBS (pH 7.2–7.4), rapidly frozen with liquid nitrogen, maintained samples at 2–8 °C after melting, added PBS (pH 7.4), homogenized by grinders, carried centrifugation 20-min at the speed of 2000–3000 r.p.m. and finally removed the supernatant.

Assays were performed in 48-well immunoplates (Chemicon International). ELISA analyses for VEGF-A and CTGF were performed using commercially available kits for rat VEGF-A (Yonghui Biotechnology Co., Ltd, Beijing, China) and rat CTGF (Yonghui Biotechnology Co., Ltd, Beijing, China) respectively. Experiments were performed according to the manufacturers’ instructions [13].

### Statistics

Statistical analyses were carried out using SPSS (version 14.0, SPSS, IL, USA). All values were expressed as mean ± SD. *P* < 0.05 was considered statistically significant. All data sets were independent, normally distributed, and with equal variance. Comparisons between multiple groups were performed by one-way ANOVA followed by Bonferroni’s post hoc test.

## Results

### Body weights and whole blood glucose levels

**Fig. 2:**
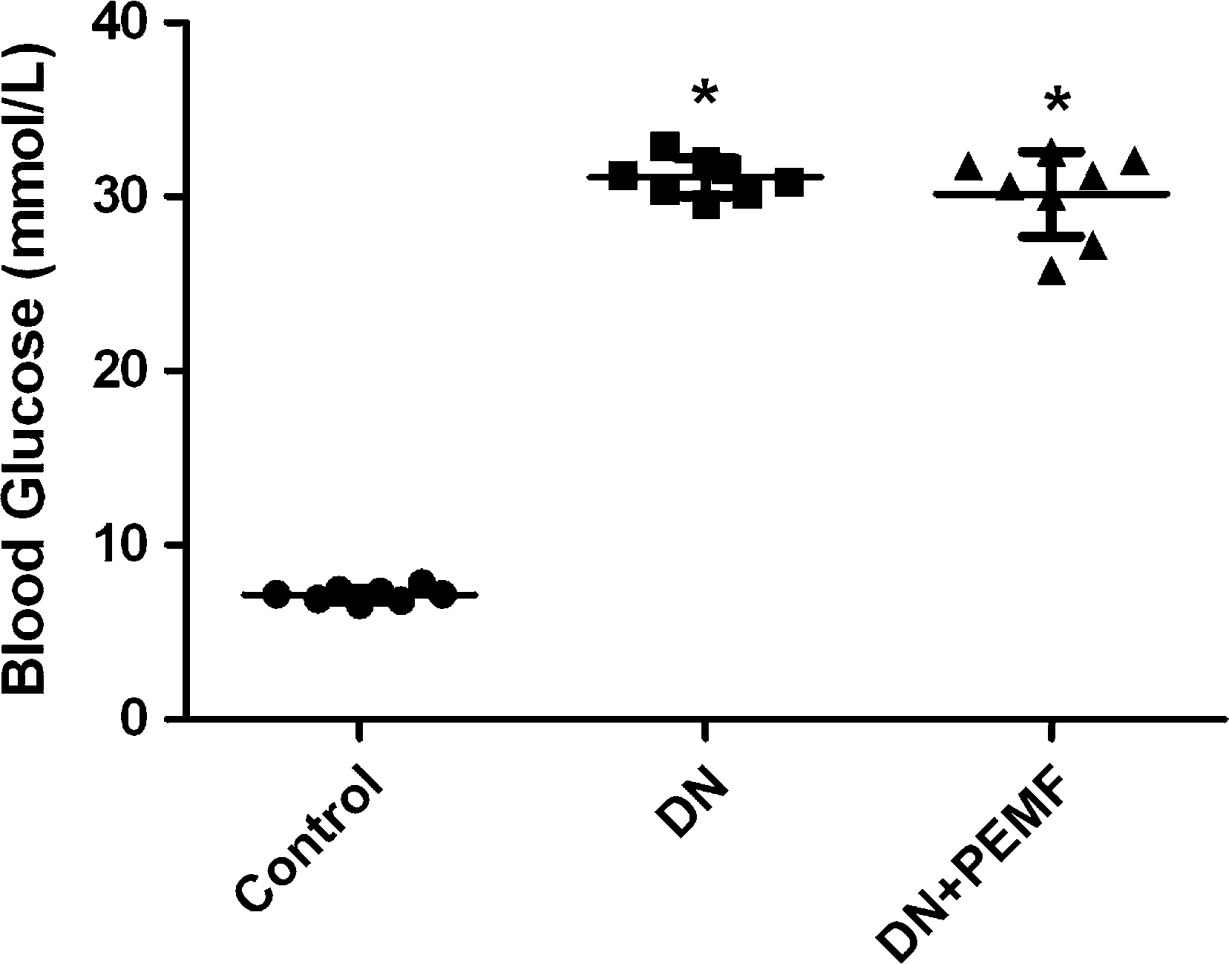
Scatter plot for weights of STZ-induced diabetic rats with DN after 6-week exposure to ELFPMF. Plot is expressed as mean ± SD (n = 8), *P < 0.05 vs Control group

**Fig. 3:**
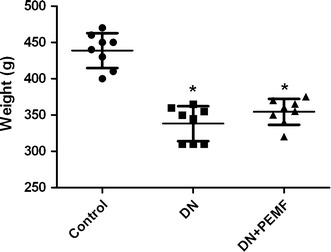
Scatter plot for blood glucose levels of STZ-induced diabetic rats with DN after 6-week exposure to ELFPMF. Plot is expressed as mean ± SD (n = 8), *P < 0.05 vs Control group

After 6-week diabetic period, as shown in Fig. 2, One-way ANOVA with Bonferroni’s post hoc determined that the mean body weight of DN group and DN + ELFPMF group were statistically significantly lower than that in the Control group (*P* < 0.05). After STZ injection, rats consistently lost weight. Although there was slightly less loss of the body weight in ELFPMF treated diabetic rats, no significant difference between DN + ELFPMF group and DN group was found (*P* > 0.05). Six weeks’ ELFPMF stimulation did not statistically significantly affect the loss of body weight caused by diabetes. Similarly, after 6-week diabetic period, one-way ANOVA with Bonferroni’s post hoc revealed that the average blood glucose levels of DN group and DN + ELFPMF group were statistically significantly higher than that in the Control group (*P* < 0.05) (Fig. 3). STZ administration caused a rapid elevation of average blood glucose levels (>27.8 mmol/L) within one week, which persisted for up to 6 weeks. Although the blood glucose levels were slightly lower in ELFPMF treated diabetic rats, there was no significant difference between DN + ELFPMF group and DN group (*P* > 0.05). Six weeks’ ELFPMF stimulation did not statistically significantly affect the hyperglycemia caused by diabetes.

### Light microscopy

**Fig. 4:**
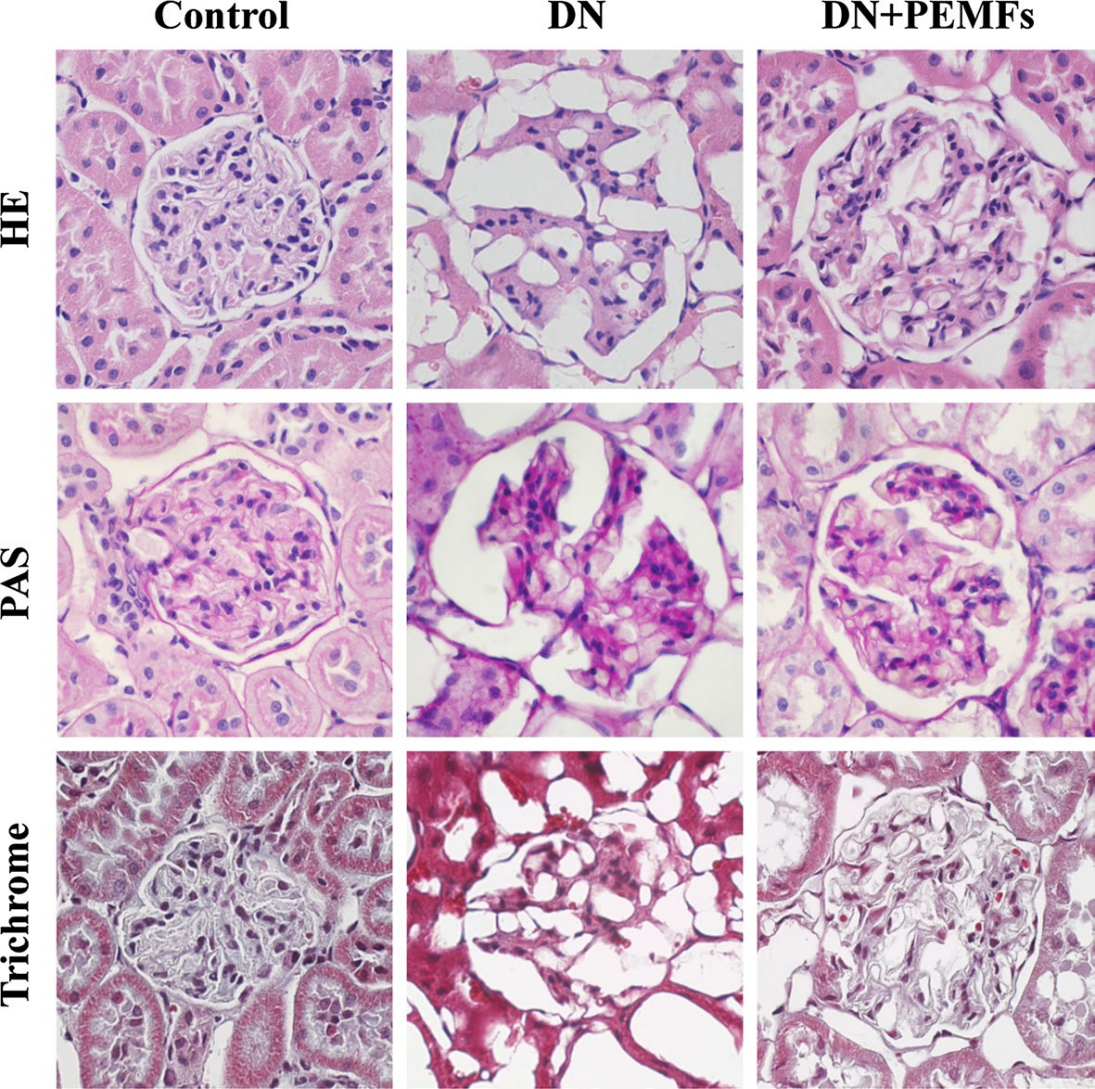
Representative glomeruli from Control, DN, and DN + ELFPMF groups after 6-week experimental period (magnification: ×400). Renal cortex sections were stained with hematoxylin-eosin (HE), periodic acid Schiff (PAS), and Masson’s trichrome

Glomerular lesions were observed in HE-stained tissue sections from STZ-injected rats at the end of the 6-week experimental period (Fig. 4, HE panel). Glomerular hypertrophy and mesangial matrix expansion were observed in the DN group at the end of the 6-week experiment period. However, these changes were ameliorated in the DN + ELFPMF group compared with DN group. Treatment with ELFPMF partially restored the normal morphology of glomeruli in diabetic rats (Fig. 4, HE panel).

Glycogen deposition in the glomeruli was measured by PAS staining to indicate the severity of glomerulosclerosis. PAS staining showed STZ-induced glomerulosclerosis, which was partially improved in ELFPMF treated diabetic rats (Fig. 4, PAS panel).

Since extracellular matrix deposition is another hallmark of glomerulosclerosis, collagen was visualized using Masson’s trichrome staining (Fig. 4, Trichrome panel). STZ treatment resulted in collagen accumulation inside glomeruli or in the periglomerular area, which was reduced in ELFPMF treated group.

These results clearly demonstrate that these diabetic rats develop glomerular hypertrophy, glomerular extracellular matrix accumulation, increasement of GBM thickness and mesangial proliferation within 6 weeks after diabetes debut. What’s more, the results from our study also revealed the ability of ELFPMF treatment to attenuate STZ-induced pathological alterations in renal structure during the progression of DN.

### Electron microscopy

**Fig. 5:**
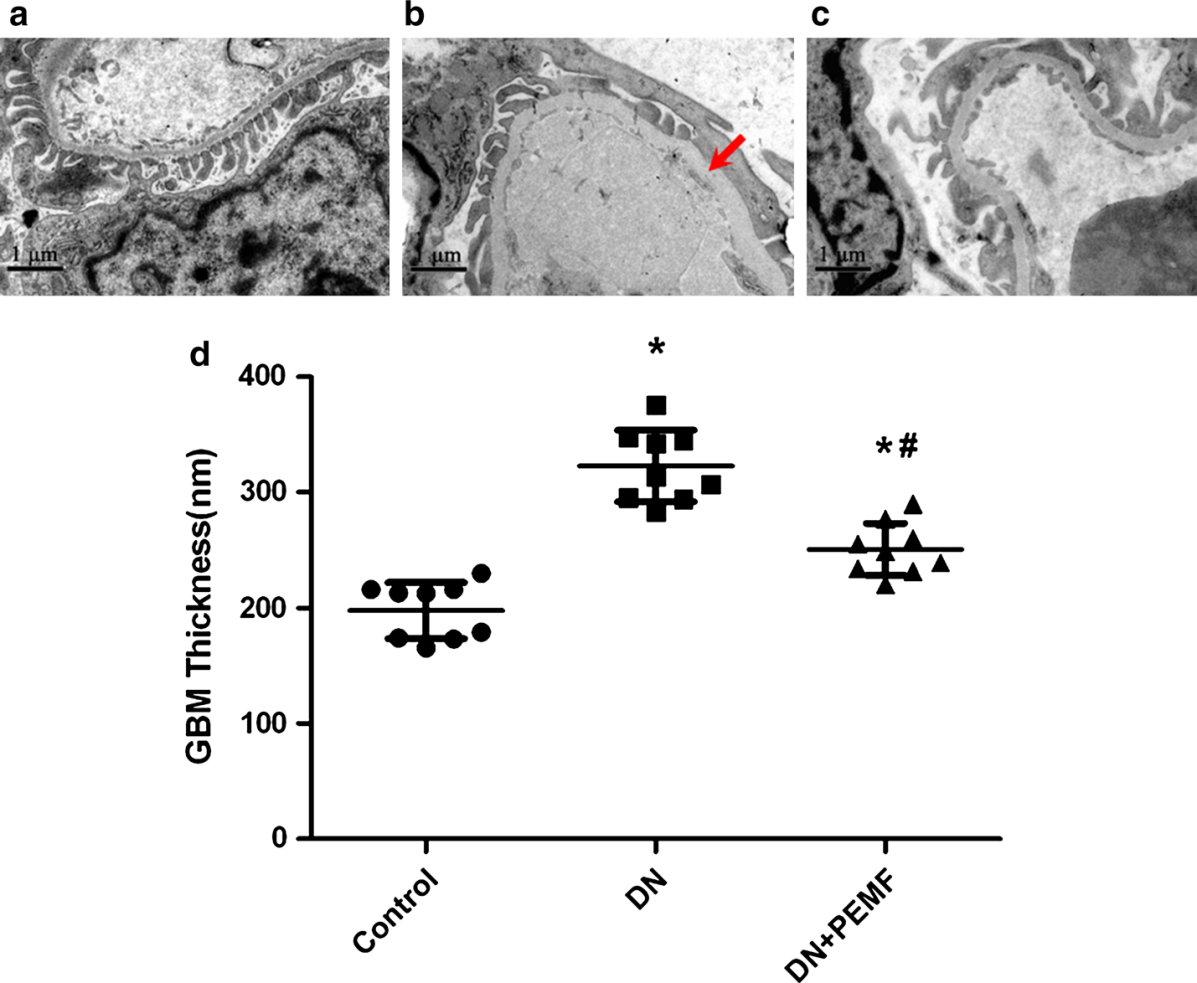
Electron microscopy of the GBM and podocyte foot processes in Control, DN, and DN + ELFPMF groups after 6-week experimental period are displayed (magnification: ×10,000). **a** Control group: glomeruli with normal structure and morphology were observed and podocyte foot processes were regularly shaped. **b** DN group: Glomeruli from diabetic rats displayed some degree of thickness of the GBM, effacement and confluence of podocyte foot processes were also observed. **c** DN + ELFPMF group: glomeruli and podocyte foot processes were abnormal, but the damage was slighter than that in DN group. **d** Scatter plot for GBM thickness in Control, DN, and DN + ELFPMF groups are plotted as mean ± SD (n = 8 per group). The mean value of GBM thickness measured at 30 different sites was considered as the GBM per rat. *P < 0.05 vs Control group, ^#^P < 0.05 vs DN group

Ultrastructural examination of glomeruli was obtained by using transmission electron microscopy after 6-week experimental period in all rats. In Control group, glomeruli with normal structure and morphology were observed and podocyte foot processes were regularly shaped (Fig. 5a). Glomeruli from diabetic rats displayed some degree of thickness of the GBM which has been an independent criterion for glomerulosclerosis (Fig. 5b, arrow, d). What’s more, effacement and confluence of podocyte foot processes were observed in DN group (Fig. 5b). In DN + ELFPMF group, glomeruli and podocyte foot processes were abnormal, but the damage was slighter than that in the DN group (Fig. 5c). Especially, the thickness of GBM was improved by 6-week ELFPMF exposure (Fig. 5d).

### Effects of ELFPMF on renal expression of VEGF-A

**Fig. 6:**
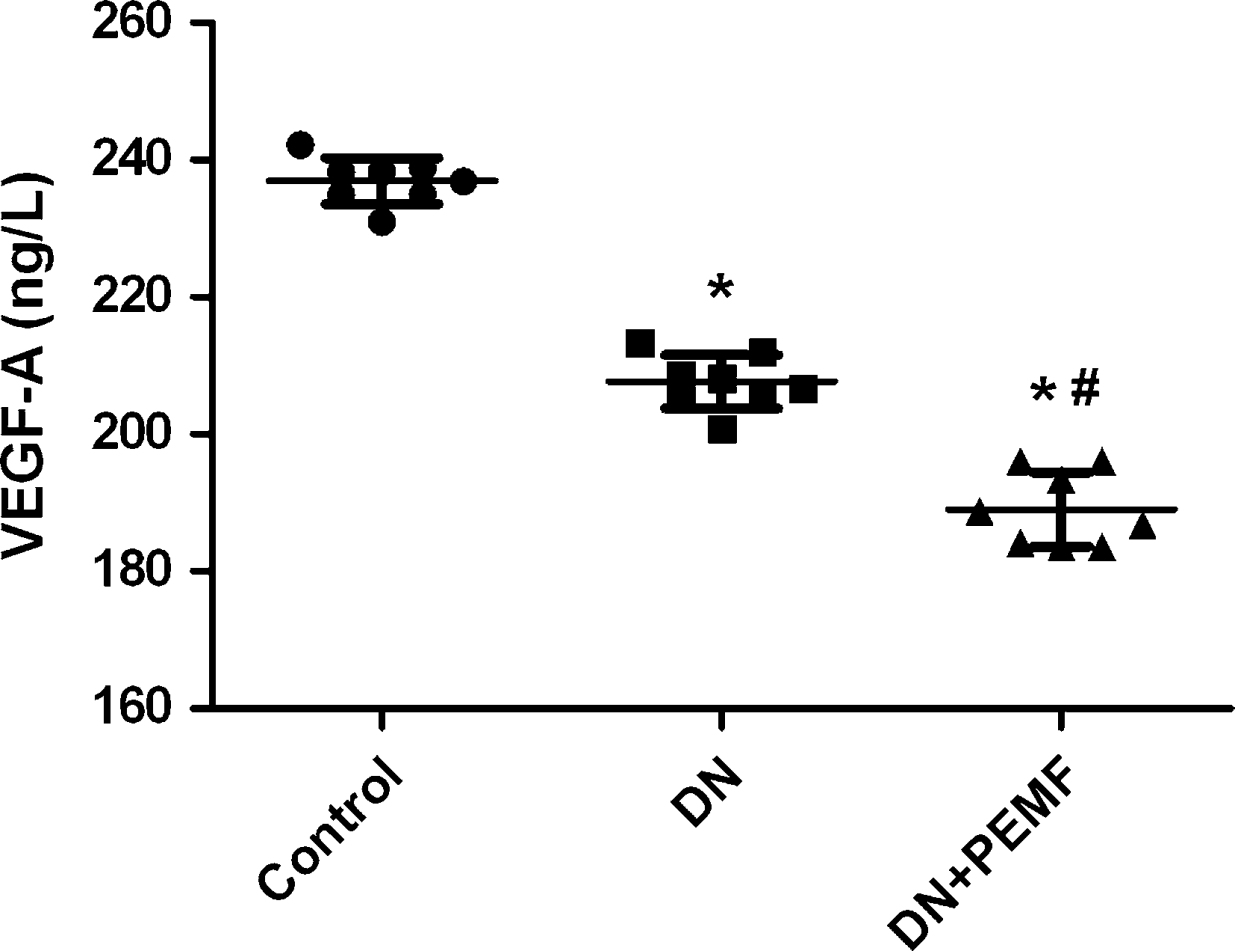
ELISA analysis for VEGF-A expression in renal cortex in Control, DN, and DN + ELFPMF groups after 6-week experimental period (n = 8). *P < 0.05 vs Control group; ^#^ *P* < 0.05 vs DM group

Bonferroni-adjusted pairwise comparisons revealed that the renal expression of VEGF-A in DN group and DN + ELFPMF group were statistically significantly lower than that in the Control group (*P* < 0.01) (Fig. 6). The renal expression of VEGF-A significantly decreased in diabetic rats compared to that in the non-diabetic rats. In addition, the renal expression of VEGF-A in DN + ELFPMF group was statistically significantly lower than that in the DN group (*P* < 0.05) (Fig. 6). ELFPMF stimulation significantly decreased the renal expression of VEGF-A in diabetic rats.

### Effects of ELFPMF on renal expression of CTGF

**Fig. 7:**
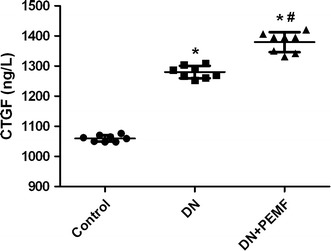
ELISA analysis for CTGF expression in renal cortex in Control, DN, and DN + ELFPMF groups after 6-week experimental period (n = 8). *P < 0.05 vs Control group; ^#^ *P* < 0.05 vs DM group

Bonferroni-adjusted pairwise comparisons revealed that the renal expression of CTGF in DN group and DN + ELFPMF group were statistically significantly higher than that in the Control group (*P* < 0.01) (Fig. 7). The renal expression of CTGF significantly increased in the diabetic rats compared to that in the non-diabetic rats. In addition, the renal expression of CTGF in DN + ELFPMF group was statistically significantly higher than that in the DN group (*P* < 0.05) (Fig. 7). ELFPMF stimulation significantly increased the renal expression of CTGF in diabetic rats.

## Discussion

In the current investigation, a marked decrease in body weight of diabetic rats was observed 6 weeks after the debut of diabetes as compared to that in non-diabetic control rats. The reduction in body weight is probably related to the osmotic diuresis and dehydration induced by diabetic hyperglycemia [14]. Meanwhile, blood glucose level rose immediately after the STZ injection, reached quite a high level at first week (>27.8 mmol/L), and then remained approximately at 27 mmol/L. The results of the current study have confirmed previous findings that blood glucose level elevated and body weight decreased in diabetic rats after STZ administration [15]. Our results also revealed that ELFPMF stimulation did not statistically significantly prevent the weight loss caused by diabetes, which is consistent to our and others’ previous investigation [10, 14]. However, contrary to the findings researched by Mert [14] and Jing [11] who observed that ELFPMF had efficacy in anti-hyperglycemia in diabetic rats, we found that the application of ELFPMF did not statistically significantly alter hyperglycemia in diabetic rats during the whole experimental period (6 weeks). The different effects of ELFPMF on hyperglycemia might be ascribed to the different standards for diabetic model adopted by us and them. The higher standards (>20 mmol/L) for diabetic model adopted by us than Mert’s and Jing’s (>16.7 mmol/L) might cause a severe damage which could not be reversed by ELFPMF.

Obviously, a prerequisite for a clear understanding of the pathophysiological mechanisms of nephropathic appearance and treatment is to know if there are definite structural changes in the kidney and to what extent they exist in the patients or experimental animal models. The pathology of DN is characterized by increased glomerular permeability to proteins and excessive extracellular matrix accumulation in the mesangium, eventually resulting in glomerulosclerosis and progressive renal impairment [3, 4]. In the present study, some evidences of pathological alterations of the glomerulus such as progressive glomerular hypertrophy, mesangial matrix expansion, thickened GBM and effacement and confluence of podocyte foot processes were observed in STZ-treated rats with DN. Similar results were also reported by other investigators [16, 17]. In addition to this, our morphological study of kidney revealed that long-term ELFPMF stimulation partially prevented or slowed the progression of DN in STZ-treated rats, which appears to be seldom reported in animal models for DN by other investigators.

Our findings confirm earlier studies on kidney biopsies from diabetes patients and other glomerular diseases in which VEGF-A expression was also decreased in the glomeruli [18]. Similarly, another study also reported that glomerular expression of VEGF-A was reduced 1 week after STZ induction [19]. It is more likely that the down-regulation of VEGF-A, results from a reduction of insulin secretion caused by streptozotocin in different disease stages [20]. What’s more, VEGF-A is normally produced by podocytes, and podocyte loss observed by ultrastructural examination of glomeruli in our study might also cause down-regulation of VEGF-A [21, 22]. However, this would seem to contradict an experimental study on diabetic rats in which VEGF-A mRNA levels in the cortex and glomeruli were increased [23]. The reason for the differences between our findings and those reported in the literature may be that the shorter duration of disease studied than theirs [24]. In addition to this, our results also revealed that ELFPMF stimulation significantly decreased the renal expression of VEGF-A in diabetic rats, and the down-regulation of VEGF-A induced by ELFPMF is similarly reported by our previous study and other investigators [10, 12].

Glomerular hypertrophy and accumulation of extracellular matrix components, which is mediated predominantly by the prosclerotic cytokines TGF-β and CTGF, are other characteristic features of DN [4]. In our study, after 6 weeks of STZ-induced diabetes, there was a significant increase in expression of CTGF in rat renal cortex. Our data on expression of CTGF in renal cortex are in agreement with previously studies which reported that overexpression of CTGF at the mRNA or protein level has previously been demonstrated in vivo in experimental diabetic rats [25] and human kidney with DN [26], as well as in cultured high glucose stimulated HK-2 cells [27]. What’s more, our results also revealed that 6-week administration of ELFPMF statistically significantly increased the expression of CTGF measured by ELISA. The effects of ELFPMF on expression of CTGF in our study are similar to other investigations which reported that ELFPMF could promote extracellular matrix production and mineralization in osteoblastlike cells [28] and accelerate extracellular matrix synthesis in connective tissue [29].

In our study, the down-regulation of VEGF-A and up-regulation of CTGF induced by 6-week ELFPMF exposure in renal cortex indicate that ELFPMF might aggravate the symptoms of DN. However, actually, 6-week exposure to ELFPMF stimulation partially prevented the development of glomeruli degeneration in STZ-treated rats with DN by using morphological examinations. Therefore, we hypothesized that although the regulation of VEGF-A and CTGF induced by ELFPMF might partially aggravate the development of DN, ELFPMF might ameliorate kidney complications induced by diabetes through other mechanisms, because the pathogenesis of DN is complex and involves multiple pathways that lead to kidney injury, including the polyol pathway [30], protein kinase C [31], advanced glycation end products [32], high glucose-induced generation of reactive oxygen species (ROS) [33], and proinflammatory cytokines. These diabetes-induced mediators interact in their adverse effects on the kidney. In addition, suppression of ROS production [34] and anti-inflammatory [35, 36] effects have been found by numerous studies. Therefore, it is not surprising that correction of any of them by ELFPMF results in amelioration of DN. Therefore, we would argue that long-term ELFPMF stimulation might ameliorate kidney complications in diabetic rats by correction of pathogenic diabetes-induced mediators, which might lead to slighter glomerular and tubulointerstitial lesions compared to those of the DN group observed in the present study.

## Conclusions

It is suggested that ELFPMF might have two kinds of effects on diabetic rats with DN. One is negative effect, which is demonstrated by statistically significantly decreased renal expression of VEGF-A and significantly increased renal expression of CTGF in diabetic rats, and the other is positive effect, which is inferred from the slighter pathological alterations in renal structure in DN + ELFPMF group than that in DN group. More importantly, the positive effect induced by ELFPMF might play a dominant role in the procession of DN in diabetic rats, and this positive effect induced by ELFPMF might result from correction of pathogenic diabetes-induced mediators. However, further research is required to elucidate the specific mechanisms for the two-sided effects of ELFPMF on DN.
